# Aquivion perfluorosulfonic superacid as an effective catalyst for selective epoxidation of vegetable oils

**DOI:** 10.1098/rsos.211554

**Published:** 2022-04-27

**Authors:** Riccardo Polese, Elisa Pintus, Luca Nuvoli, Monica Tiana, Salvatore Pintus, Giuseppe Satta, Andrea Beccu, Silvia Gaspa, Massimo Carraro, Lidia De Luca, Ugo Azzena, Luisa Pisano

**Affiliations:** Dipartimento di Chimica e Farmacia, Università degli Studi di Sassari, Via Vienna 2 07100, Sassari, Italy

**Keywords:** epoxidation, acidic ion exchange resin catalysts, aquivion, *q* ^1^HNMR, vegetable oils, waste cooking oils

## Abstract

The acid-promoted epoxidation of vegetable oils was studied using a variety of acidic ion exchange resins as heterogeneous acid catalysts. Quantitative and selective epoxidation of a series of vegetable oils with different composition of saturated, mono-, di- and tri-unsaturated fatty acids was obtained upon identification of the more efficient catalyst and experimental conditions. Furthermore, optimized reaction conditions were successfully applied to the epoxidation of a waste cooking oil, thus extending our procedure to the valorization of a biowaste, an area of increasing importance within a more sustainable society. The use of quantitative ^1^HNMR besides making accurate evaluation of the amounts of reagents to be employed and of the selectivity, allowed facile and rapid quantification of mono-, di- and tri-epoxides, thus providing an indirect indication on the fatty acid composition of the vegetable oils, even in the presence of very low quantities of linolenic acid.

## 1. Introduction

Looking for alternative sources of raw materials with an environmentally friendly approach, biomass valorization is one of the most promising fields of investigation [1–3].

Among the valuable components of biomass, vegetable oils represent an interesting renewable feedstock for several reasons, including their everywhere availability, inherent biodegradability, low cost and their benign environmental properties [4–6]. Currently, focus should be concentrated on non-edible vegetable oils, microalgal oils and used cooking oils, as they do not suffer from excessive costs and/or competition for food purposes [7]. Additionally, the tremendous global waste cooking oils (WCOs) production, today estimated as at least 42 million tons per year, requires the set-up of efficient processes [8]. Transformation of WCOs into biofuels is a primary valorization route, although, without specific regulations, the production of these derivatives will not be, in the long term, economically sustainable [9,10]. Hence the arising interest in their transformation into added-value products.

Vegetable oils are mainly mixtures of esters of glycerol with fatty acid chain with 0–3 double bonds per carbon chain. One of the most fruitful routes of valorization is their transformation into epoxides due to current and potential commercial applications of the obtained non-toxic epoxy products. In the more recent applications, epoxidized oils are used as natural, renewable, non-toxic, non-corrosive biolubricants [11], shape memory materials [12], thermosets [13], biodegradable plasticizers [14] and stabilizers, to improve flexibility and elasticity, and to enhance the stability of polymers towards heat and UV radiation [15]. In the majority of cases, the properties of these products are related to the amount of epoxy group present in the molecule oil, frequently expressed as oxirane oxygen content or oxirane number ($g_{O_{2}}/g_{\mathrm{sample}}$). Owing to the high reactivity of the oxirane ring, these derivatives also act as raw materials for a variety of biochemicals, such as alcohols, glycols, alkanolamines, carbonyl compounds, olefinic compounds and polymers like polyesters, polyurethane (PU) and epoxy resins [16]. Epoxidized vegetable oils are also promising materials for the production of non-isocyanate polyurethanes [17–19].

The main method for the production of epoxidized vegetable oils is the Prilezhaev epoxidation (current industrial process) [20], which consists of a biphasic system with an oxidant agent (mainly hydrogen peroxide), an oxygen carrier (percarboxylic acid) and the unsaturated vegetable oil under acid catalysis [21,22]. The main drawbacks of the reaction are related to the immiscibility of reactants (oil and aqueous hydrogen peroxide) that slows down the mass transfer, the high exothermicity [23] of reaction and the selectivity often compromised by the high reactivity of the epoxy ring in the presence of acid catalysts.

Nowadays, the set-up of green epoxidation process points to replace mineral homogeneous acids with the more sustainable chemoenzymatic [24] or heterogeneous catalytic systems [25,26].

Process intensification technologies, based on the development of cleaner, safer and more energy-efficient processes, compared with conventional ones, has been recently applied to the epoxidation of vegetables oils [27]. In this context, acidic ion exchange resins (AIERs) catalysis was introduced to overcome the drawbacks related mainly to the degradation of the oxirane rings, thus improving the overall selectivity [28].

**Figure 1.**
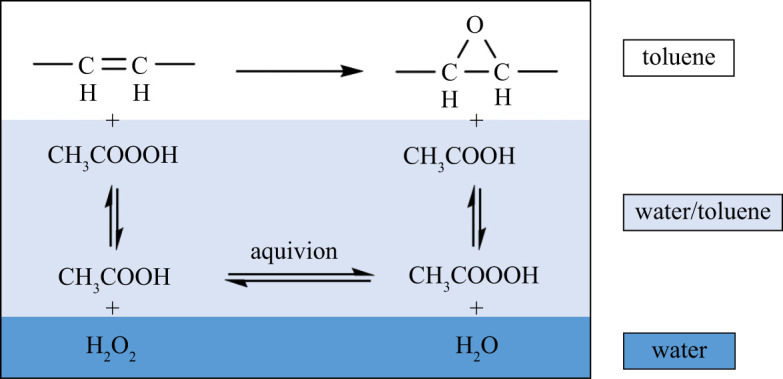
Schematic diagram of the epoxidation reaction Aquivion-catalysed with ${\mathrm{CH}}_{3}\mathrm{COOOH}$ generated *in situ* from ${\mathrm{CH}}_{3}\mathrm{COOH}$ and aqueous $H_{2}O_{2}$, and mass transfer between phases.

However, it must be considered that due to the high viscosity, in the biphasic Prilezhaev epoxidation of the vegetable oils the poor mixing cause heat and mass transfer limitations. In this context, we decided to use, besides several heterogeneous acidic catalyst of the sulfonated polystyrene type (Amberlyst 15, Amberlite IR120 and Dowex 50WX2), the commercially available perfluorosulfonic acid (PFSA) resin Aquivion PW79S, relying on its capability to increase the mass transfer rate in the peracid formation step and the heat transfer during the epoxidation by improving the liquid–liquid contact in the biphasic reaction mixture. Indeed, Aquivion PFSA is a superacid amphiphilic polymer, capable of stabilizing Pickering-like emulsions in the presence of chemicals with opposite polarities, which has been successfully used as catalyst in biphasic reaction (figure 1) [29].

Accurate set-up of reaction stoichiometry requires an exact evaluation of the fatty acid composition of oils, usually determined by gas chromatography (GC) technique, the official method of the American Oil Chemists’ Society (AOCS; [30] p. 3, line 119). However, this method requires time and work-consuming conversion of triglycerides into their more volatile methyl esters, purification, identification and GC quantification. In addition, problems related to sample oxidation during this derivatization processes have been reported [31,32]. In the last 20 years, there has been a growing interest in employing spectroscopic techniques that do not require derivatization. Among them quantitative ^1^HNMR (*q* ^1^HNMR) spectroscopy offers many advantages over other spectroscopic techniques: it is simple and rapid and a large amount of information can be extracted directly from the sample under investigation, without any kind of pre-treatment or chemical modification [33]. We performed the characterization of vegetable oils and the complete analysis of their epoxidized derivatives, as well as the epoxidation state evolution, by *q* ^1^HNMR. In what follows, we report for the first time on the catalytic performance in the Prilezhaev epoxidation of vegetable oils of Aquivion, a solid perfluorosulfonic superacid. Quantitative ^1^HNMR spectroscopy was employed as a practical and robust analytical tool to evaluate its catalytic efficiency, also in comparison with different types of AIERs whose catalytic activity in this reaction was already reported.

## 2. Material and methods

### 2.1. Materials

Glacial acetic acid (min. 99.5–99.9 wt%) and hydrogen peroxide (30 wt%), were purchased from Sigma Aldrich. Ion-exchange resins Amberlyst^®^ 15, Amberlite^®^ IR-120 (H) and Aquivion^®^ PW79S (purchased from Sigma-Aldrich) and Dowex^®^ 50WX2 (H) (purchased from Alfa Aesar) were used as received. Safflower and thistle refined seed oil were kindly provided by Novamont S.p.A. Hemp oil was bought at a local store. Waste cooking oil (WCO) was obtained by a local company, Il Gabbiano S.r.l. All the other reagents used were of analytical grade and were purchased from VWR.

### 2.2. Epoxidation procedure

The epoxidation of safflower oil is illustrative of a general procedure. The reactions were carried out in a 100 ml two-neck spherical glass reactor (internal maximum diameter 60 mm) equipped with an octahedral magnetic stirrer bar (length 15 mm, diameter 8 mm), a temperature probe and reflux condenser under air atmosphere. In a typical experiment to a solution of 5 g of oil (5.6 × 10^−3^ mol, $28.2\times 10^{-3}\;C=C\;\mathrm{mol}$) in 10 ml of toluene were added 1.6 ml of glacial acetic acid (28.2 × 10^−3^ mol) and the calculated amount of AIER catalyst (see tables 1 and 2); the mixture was then conditioned at 0°C for 5 min. Next, under stirring, 8.4 ml of hydrogen peroxide (30% w/w, 8.2 × 10^−2^ mol) was added drop-wise during 10 minutes, thus leading to a $C=C/H_{2}O_{2}/{\mathrm{CH}}_{3}\mathrm{COOH}\;\mathrm{molar}\;\mathrm{ratio}=1\;:\;3\;:\;1$. Once the addition was finished, the mixture was stirred at 1400 r.p.m. (onset of the vortex at 600 r.p.m.) and heated at 60°C for 6 h. The catalyst was filtered away and the biphasic solution separated. The organic phase was washed with saturated sodium bicarbonate (10 ml), saturated sodium chloride (10 ml) and water (10 ml). The resulting organic phase was then dried with anhydrous ${\mathrm{MgSO}}_{4}$ and, after filtration, distilled under vacuum to remove the toluene.

**Table 1.** Catalytic epoxidation of safflower oil promoted by AIER catalysts.^a^

exp.	AIER catalyst	$\mathrm{meq}\;H^{+b}$	conv. (%)	yield (%)	selectivity (%)	TOF (h^−1^)^c^
1	$H_{2}{\mathrm{SO}}_{4}$	60	99.8	94.2	94.4	
2	Amberlite® IR-120 (H)	33	50.7	48.6	95.9	2.42
3	Amberlite® IR-120 (H)	44	70.6	67.5	95.6	
4	Amberlite® IR-120 (H)	128	98.6	94.7	96.0	
5	Amberlyst® 15	33	36.6	33.8	92.4	2.42
6	Amberlyst® 15	44	55.8	53.5	95.8	
7	Amberlyst® 15	150	90.1	87.3	96.9	
8	Amberlyst® 15	170	95.3^d^	87.0	91.3	
9	Dowex® 50WX2 (H)	33	87.8	82.8	94.4	4.05
10	Dowex® 50WX2 (H)	66	99.5	94.2	94.6	
11	Aquivion® PW79S	17	85.4	79.4	93.0	8.03
12	Aquivion® PW79S	22	94.5	89.0	94.1	
13	Aquivion® PW79S	27	99.6	91.8	92.2	
14	Aquivion® PW79S	33	99.9	91.3	91.4	
15	Aquivion® PW79S	66	99.5	90.9	91.4	
16	Aquivion® PW79S	126	99.9	88.1	88.2	

^a^The epoxidation was performed in toluene for 6 h at 60°C; ratio $C=C/H_{2}O_{2}/{\mathrm{CH}}_{3}\mathrm{COOH}\;1\;:\;3\;:\;1$.

^b^ $\mathrm{meq}\;H^{+}=H^{+}/C=C$ molar ratio.

^c^ $\mathrm{TOF}=\mathrm{mol}\;\mathrm{epoxide}/(\mathrm{mol}\;H^{+}\times \mathrm{time})\;(h^{-1})$.

^d^Quantitative conversion yields were obtained performing the reaction for 24 h.

**Table 2.** Catalytic epoxidation of different vegetable oils.

exp.	vegetable oil	*DB*_*m*_	catalyst (meq $H^{+}$)^a^	conv. (%)	yield (%)	selectivity (%)
1	safflower	5.03	Aquivion (33)	100.0	92.1	92.1
2	thistle	4.34	Aquivion (33)	99.9	94.3	94.4
3	WCO	3.92	Aquivion (33)	98.6	93.3	94.7
4	hemp	5.81	Aquivion (66)	99.7	91.07	91.3
5	safflower	5.03	Dowex (33)	87.8	82.8	94.4
6	hemp	5.81	Dowex (66)	94.9	89.1	91.3

^a^$\mathrm{meq}\;H^{+}=H^{+}/C=C$ molar ratio.

### 2.3. Quantitative ^1^HNMR methods

**Figure 2.**
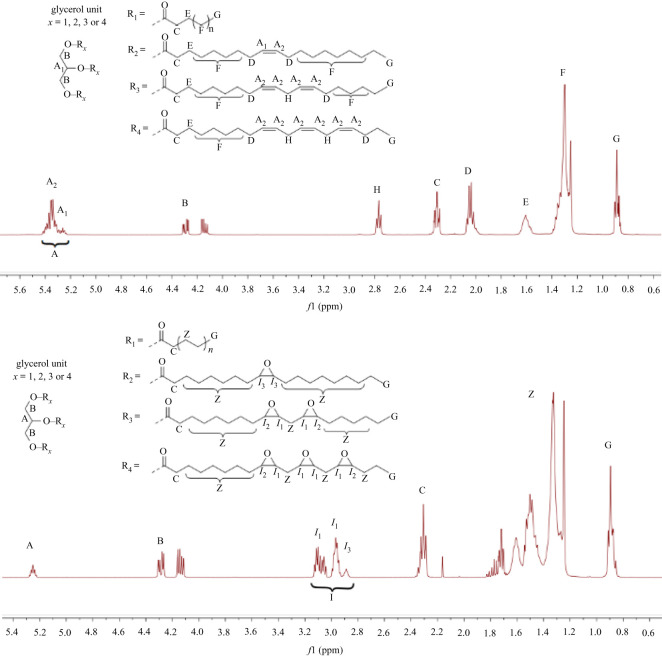
*q* ^1^HNMR spectra of safflower oil and epoxidated safflower oil (NMR 400 MHz, ${\mathrm{CDCl}}_{3}$).

**Figure 3.**
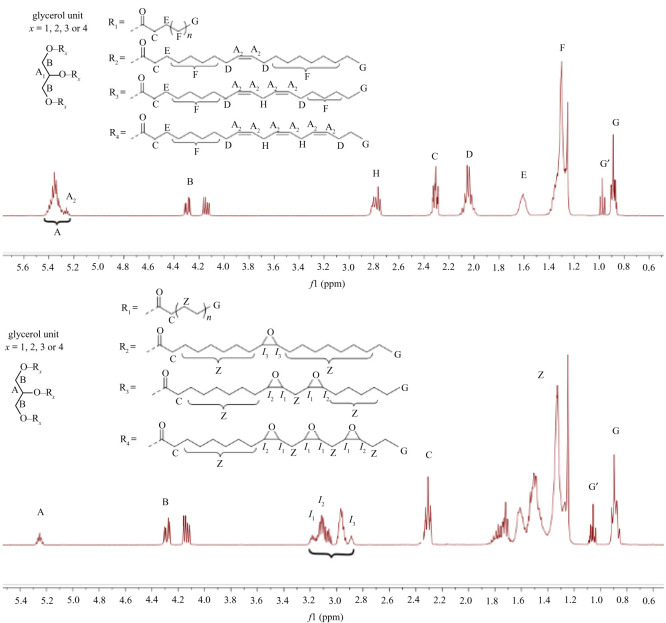
*q* ^1^HNMR spectra of hemp oil and epoxidated hemp oil (NMR 400 MHz, ${\mathrm{CDCl}}_{3}$).

Vegetable oils and epoxidized vegetable oils were characterized by *q* ^1^HNMR (electronic supplementary material). As examples, in figure 2 are given the *q* ^1^HNMR spectra of the safflower oil, with a very low content of linolenic acid, and its epoxidized derivative, and in figure 3 are given the *q* ^1^HNMR spectra of the hemp oil, with a relevant content of linolenic acid, and its epoxidized derivative. The *q* ^1^HNMR spectra were recorded on a Bruker Avance 400 MHz spectrometer and the signal of chloroform was used as reference for the chemical shift values. The following acquisition parameters have been used: 45 pulse angle of 4.87 μs pulse width; transmitter frequency offset (O1P) of 5 ppm; relaxation delay (d1) time 10 s; acquisition time 2.05 s; spectral width 18.75 ppm, 72 scans; spectral acquisition temperature 291–298 K. All samples were prepared by dissolving 15 mg of sample in 0.6 ml of ${\mathrm{CDCl}}_{3}$ in a 5 mm diameter NMR tube. In the *q* ^1^HNMR experiments, to avoid differential saturation effects, the recycling time, i.e. acquisition time + relaxation delay, was calibrated on the higher T1 values, according to the Ernst equation [34]. Hence, proton T1 relaxation time were previously calculated on each ^1^HNMR signal of the safflower oil and of the epoxidized safflower oil, by using the standard inversion recovery T1 pulse sequence (electronic supplementary material) as provided in the Brucker software NMR TopSpin (3.1); the corresponding T1 values calculated with the MestReNova software (6.0.2–5475) are reported in table 3.

**Table 3.** Longitudinal relaxation times T1 of proton nuclei of the safflower oil and its epoxidized derivative.

ppm (${\mathrm{CDCl}}_{3}$)	substrate	proton assignment^a^	peak notation^b^	T1 (s)	integral limits (ppm)
5.35	safflower oil	CH=CH CH gly	A	2.06	5.46–5.21
4.28	safflower oil	2xCH gly	B1	0.52	4.37–4.23
4.13	safflower oil	2xCH gly	B2	0.54	4.21–4.08
2.77	safflower oil	${\mathrm{CH}}_{2}\;\mathrm{bisallylic}$	H	1.52	2.85–2.70
2.31	safflower oil	${\mathrm{CH}}_{2}\;\alpha -\mathrm{CO}$	C	0.84	2.39–2.23
2.04	safflower oil	${\mathrm{CH}}_{2}\;\mathrm{allylic}$	D	1.33	2.12–1.96
1.61	safflower oil	${\mathrm{CH}}_{2}\;\beta -\mathrm{CO}$	E	0.84	1.69–1.53
1.30	safflower oil	$-{\mathrm{CH}}_{2}-$	F	1.11	1.43–1.21
0.89	safflower oil	$-{\mathrm{CH}}_{3}$	G	2.65	0.95–0.82
5.26	safflower epoxide	CH=CH, CH gly	A	1.04	5.31–5.22
4.29	safflower epoxide	2xCH gly	B1	0.49	4.38–4.22
4.15	safflower epoxide	2xCH gly	B2	0.49	4.22–4.08
3.10	safflower epoxide	tri- + di- epox	I1	1.91	3.20–3.03
2.98	safflower epoxide	di- + tri- epox	I2	1.73	3.03–2.94
2.89	safflower epoxide	mono epox	I3	1.86	2.94–2.86
2.32	safflower epoxide	${\mathrm{CH}}_{2}$ *α* −CO	C	0.76	2.43–2.24
1.54	safflower epoxide	${\mathrm{CH}}_{2}$ *β* −CO, $-{\mathrm{CH}}_{2}-$	Z	0.87	1.90–1.19
0.90	safflower epoxide	$-{\mathrm{CH}}_{3}$	G	2.16	0.98–0.82

^a^As previously reported by Xia *et al.* [35].

^b^The signals letters agree with those in figures 2 and 3.

The ^1^HNMR signals of our vegetable oils [36], as well as of their epoxidized derivatives have been assigned accordingly with what previously described [35,37]. All spectra were analysed by the MestReNova package, using the processing protocol optimized for *q* ^1^HNMR analysis [38]. Integration of each spectrum was performed thrice after manual phase and the fifth-order baseline correction. Integral values were normalized by assigning two protons to the signal of the diasterotopic methylenic protons of the glycerolic portion appearing at 4.3 ppm. This signal was chosen as internal standard and used as normalization factor (NF), since it is stable and clean during the epoxidation of the vegetable oils investigated and for its low T1 value (table 4, equation (1)). The use of *q* ^1^HNMR besides making accurate control of epoxidation stoichiometry is a valuable tool for determining conversions, yields, selectivities and oxirane oxygen content. Expanding the ^1^HNMR analytical methodology developed by Miyake *et al.* [39,40] we have performed, through the equations (1)–(14), the analytical calculation of several chemical proprieties of the studied oils and of their epoxidized derivatives allowing to accurately control the stoichiometry and the performances of the epoxidation reactions (detailed *q* ^1^HNMR analysis of vegetable oils and epoxidized vegetable oils are reported in the electronic supplementary material). In the presence of unimportant amounts of mono-, di-glycerides, trans-fatty acids and free fatty acids [41], just processing the *q* ^1^HNMR spectra we were able to determine, with an accuracy comparable to that of the official method (GC-FID) the most important chemical properties. Moreover, the equations allow to analyse vegetables oils containing relevant (hemp oil) or irrelevant amount of tri-unsaturated fatty acids (safflower oil). Based on the integrals of each *q* ^1^HNMR signal (figures 2 and 3) normalized as previously described, we have calculated the mean molecular weight of the triacylglycerides (TAG MWm) and of its epoxidized derivative (EPOX MWm) according to equations (2) and (3), respectively; the oxirane oxygen content and the mean double-bond content by equations (4) and (5), respectively; the mono-, di-and tri-unsaturated free acid content can be easily determined by equations (6)–(11).

**Table 4.** *q* ^1^H NMR integral relationship for composition determination.

name	relationship	
normalizing factor	$\mathrm{NF}=\frac{B}{2}$	(1)
triacylglycerides mean molecular weight	TGA MW_*m*_ = 15.034 ∗ (*G* + *G*′)/3NF + (14.026 ∗ [*C* + *D* + *E* + *F* + (*H* − (4/3) ∗ *G*′) + (2 ∗ (*G*′/3))])/2NF + (173.100 ∗ *B*/2NF) + (26.016 ∗ [*A* − NF]/2NF)	(2)
epoxidated derivative mean molecular weight	EPOX MW_*m*_ = (15.034 ∗ (*G* + *G*′)/3NF) + (14.026 ∗ [*C* + *Z*]/2NF) + (173.100 ∗ *B*/2NF) + (42.015 ∗ *I*/2NF) + (26.016 ∗ (*A* − NF)/2NF)	(3)
oxirane oxygen	$(g_{O_{2}}/g_{\mathrm{sample}})=(\frac{I}{2}\;*\;15.999/{\mathrm{EPOX}\;\mathrm{MW}}_{m})\;*\;100$	(4)
mean double-bond content	DB_*m*_ = (*A* − 1)/2	(5)
oil polyunsaturated fatty acids	$\text{\%}\mathrm{PUFA}=(\left[(H-\frac{4}{3}G^{\prime })+(\frac{2}{3}G^{\prime })\right]/C)\;*\;100$	(6)
oil monounsaturated fatty acids	%MUFA=(D/2C) ∗ 100−PUFA	(7)
oil unsaturated fatty acids	%UNS=(D/2C) ∗ 100	(8)
oil saturated fatty acids	%SFA=100−UNS	(9)
oil tri-unsaturated	$\text{\%}\mathrm{TRI}=\frac{\frac{2G^{\prime }}{3}}{C}\;*\;100$	(10)
oil di-unsaturated	$\text{\%}\mathrm{DI}=\frac{H-\frac{4G^{\prime }}{3}}{C}\;*\;100$	(11)
MonoEpox	$\text{\%}\mathrm{MonoEpox}=\frac{\left(I_{3}/2\right)}{\left(I_{3}/2)+(I_{2}-(I_{1}/2))+((I_{1}/2)-(I_{2}/2)\right)}\;*\;100$	(12)
DiEpox	$\text{\%}\mathrm{DiEpox}=(I_{2}-(I_{1}/2)/\left((I_{3}/2)+(I_{2}-(I_{1}/2))+((I_{1}/2)-(I_{2}/2))\right))\;*\;100$	(13)
TriEpox	$\text{\%}\mathrm{TriEpox}=(\left(I_{1}/2)-(I_{2}/2\right))/(\left(I_{3}/2)+(I_{2}-(I_{1}/2))+(I_{1}/2-(I_{2}/2)\right))\;*\;100$	(14)

In the presence of low amounts of tri-unsaturated fatty acid chains, the fatty acids composition can be more accurately determined by table 4 equations (12)–(14) based on the values of the integrals of the three signals of the epoxy groups *I*_1_, *I*_2_ and *I*_3_ [35].

## 3. Result and discussion

### 3.1. Prilezhaev epoxidation

Taking into consideration the findings of previous studies, we have planned to optimize in terms of yields and selectivity the Prilezhaev epoxidation of vegetables oils under AIERs catalysis. To this aim, we have developed a methodology able to convert quantitatively and selectively the vegetable oils into epoxides with a safe method, regardless of their fatty acid compositions. Indeed, it is known that the oxirane oxygen content is a determining factor for the numerous useful applications of these derivatives; furthermore, the safety factor should play a key role in the success and scalability of any chemical process. To catalyse the perhydrolysis reaction (*in situ* peracid formation from carboxylic acid and $H_{2}O_{2}$), we used some of the most efficient AIERs epoxidation catalysts [28,42]: Amberlite^®^ IR-120 (H), Amberlyst^®^ 15, Dowex^®^ 50WX2 (H) and, for the first time, the perfluorosulfonic superacid Aquivion^®^ PW79S. Aquivion is a Pickering interfacial catalyst with combined superacid and amphiphilic properties, thus capable to promote acid-catalysed biphasic reaction by improving mass-transfer of the reagents to the active sites under mild conditions. In the presence of formic acid, the reaction can be performed without the addition of acid catalysts. However, for safety problems, we have preferred to use acetic acid instead of formic acid, which is more corrosive and toxic [43]. Acetic acid is also preferable, in a comparison with formic acid, for its lower cost of regeneration; furthermore, performic acid creates greater risks of explosive decomposition if it is used in conditions of high concentrations and high temperatures [44]. Elsewhere, as a good compromise, we fixed 1 : 1 as its molar ratio with respect to the double-bond moles; indeed, low acid concentration can compromise the oxygen transfer to the oil or significantly increase reaction times and therefore energy depletion, while its high concentration can promote the cleavage of the oxirane rings [37].

Although the AIER catalysts are normally easily recycled, we have evaluated carefully the lowest amount necessary to promote quantitative epoxidation; indeed, it is expected that an increase in the catalyst amount accelerates the reaction rate, and thus the rate of energy release. For the same reasons, we also preferred to use the hydrogen peroxide at a lower concentration value as a safer alternative to the more concentrated reagent. Finally, an intermediate temperature (60°C) between the most used (35–80°C), was selected to ensure a good selectivity within a reasonable time (6 h). Indeed, long reaction times can promote side reactions and results in wasted energy. Initially, some experiments have been carried out without any added co-solvent; however, under these environmentally friendly conditions, we observed formation of by-products before the complete conversion of the C=C double bonds. Therefore, we decided to perform the epoxidation reactions in the presence of an organic co-solvent, not only to minimize the formation of these by-products [45], but also to reduce the viscosity of the reaction mixtures and obtain an efficient dispersion of the reaction heat (Δ*H* = 230.3 kJ mol^−1^ for each double bond) [22]. After a thorough screening, we decided to employ toluene as a co-solvent, both due to its relatively high boiling point and inertness toward the formation of peroxides. Indeed, these dangerous by-products formed in not negligible quantities in reactions run in the presence of solvents with a lower environmental impact such as CPME and AcOtBu, as determined by a commercially available kit (Quantofix^®^, measuring range 0.5–25 mg l^−1^ $H_{2}O_{2}$), allowing a semi-quantitative evaluation of peroxides.

We have tested divinylbenzene-based resins with different degree of cross-linking to catalyse the Prilezhaev epoxidation of safflower oil, chosen as model substrate of vegetable oils (table 1). Great attention has been paid to the equivalent of $H^{+}$ employed ($H^{+}$/double-bond molar ratio), either to control the stoichiometry of the epoxidation or to estimate the catalysts activity.

The parameters of epoxidation reaction have been calculated using *q* ^1^HNMR integral values (table 3) according to the following equations:

3.1 $$\mathrm{Conversion}=\left[100-\frac{\left(A_{\mathrm{oil}}-\mathrm{NF}\right)}{A_{\mathrm{epox}}-\mathrm{NF}}\right]\times 100$$

3.2 $$\;\;\mathrm{Yield}=\left[\left(\frac{I}{2DB_{m}}\right)/\frac{G}{9}\right]\times 100$$

3.3 $$\;\mathrm{and}\;\;\;\;\;\mathrm{Selectivity}=\frac{\mathrm{Yield}}{\mathrm{Conversion}}\times 100,$$

where *DB*_*m*_ is the mean double bonds (table 4, equation (5)).

### 3.2. Efficiency of AIER catalysts

The Prilezhaev epoxidation of vegetable oils is heavily affected by the degree of cross-linking of employed AIERs: with high cross-linking catalysts the exposure of oxiranic rings to acidic sites can be minimized, thus preventing the collateral ring opening reaction, although the rate of reactions may be slower, and in some cases, the catalyst may be deactivated [46]. For its intermediate cross-linking degree (table 5), Amberlite IR120 (4.7 meq [H^+^]/g) is considered one of the best heterogeneous catalysts for this reaction [42]. Under optimized conditions (128 meq. of $H^{+}$), with this catalyst, we obtained a good result both in yield and selectivity (table 1 entry 4). In the presence of smaller amounts of catalyst, partial conversion of the substrate was observed but the selectivity was always excellent (table 1, entries 2 and 3). These results (table 1, entries 2–4) in conjunction with the experiment carried out with 60 meq. of the homogeneous catalyst $H_{2}{\mathrm{SO}}_{4}$ (table 1 entry 1), have been used as a benchmark for any other catalyst employed in this work.

**Table 5.** Properties of AIER catalysts.^a^

catalyst	matrix	moisture	acidity meq H^+^/*g* (dry product)
Amberlyst® 15	styrene+20% divinylbenzene (macroreticular)	52–57%	4.7
Amberlite® IR-120 (H)	styrene+8% divinylbenzene (macroreticular)	53–58%	4.5
Dowex® 50WX2 (H)	styrene+2% divinylbenzene (gel)	80%	4.3–4.8
Aquivion® PW79S	fluoroethylene + sulfonyl fluoride vinyl ether	0–2%	1.2–1.3

^a^Data from datasheet available online from suppliers; more information is also accessible.

By using Amberlyst® 15 (4.7 meq [H^+^]/g), with the same acidity but higher cross-linking (table 5), we observed, as expected, in 6 h good selectivity but poor conversion yields (table 1, entries 5–7). Better result with this catalyst has been obtained by using 170 meq. of $H^{+}$ and increasing the reaction time to 24 h; however, under these conditions, a slight decrease in selectivity was observed (table 1, entry 8). Excellent results, in conversion, yield and selectivity, were obtained by using the resin Dowex® 50WX2 (H) with similar acidity (4.6 meq [H^+^]/g) and a lower degree of cross-linking (table 1, entries 9 and 10). With this catalyst, we obtained complete conversion and excellent selectivity by employing an amount of catalyst corresponding to 66 meq. of $H^{+}$ (table 1, entry 10). However, the most interesting results were obtained by employing the Aquivion superacid resin with an acidity comparable to the value of pure sulfuric acid. This solid perfluorinated superacid catalyst is commercialized as coarsely grained white powders as suitable ready to use with an acid capacity between 1.0 and 1.3 meq g^−1^, is resistant in highly aggressive environments with a very high thermo-mechanical stability, compatible with water and many organic solvents, safe and easy to handle, easy to recover from a liquid phase [47]. Among the commercially available products, we have selected the powder Aquivion PFSA PW79S with the higher acid capacity (1.23−1.30 meq g^−1^) and an excellent swelling. In the first experiments, we have employed an amount of Aquivion PW79S like that used with the other catalyst obtaining quantitative conversion with a slight decrease in selectivity (table 1, entries 14–16) with respect to the better selectivity obtained with the other catalysts employed. Thereafter, we optimized the reaction conditions by assessing the lowest amount of catalyst necessary to obtain quantitative conversion of the double bonds into oxiranes (table 1, entries 13–14). Under optimized reaction conditions, we obtained a quantitative conversion by employing an amount of catalyst corresponding to 33 meq. of $H^{+}$, maintaining an excellent selectivity (table 1, entry 14).

**Figure 4.**
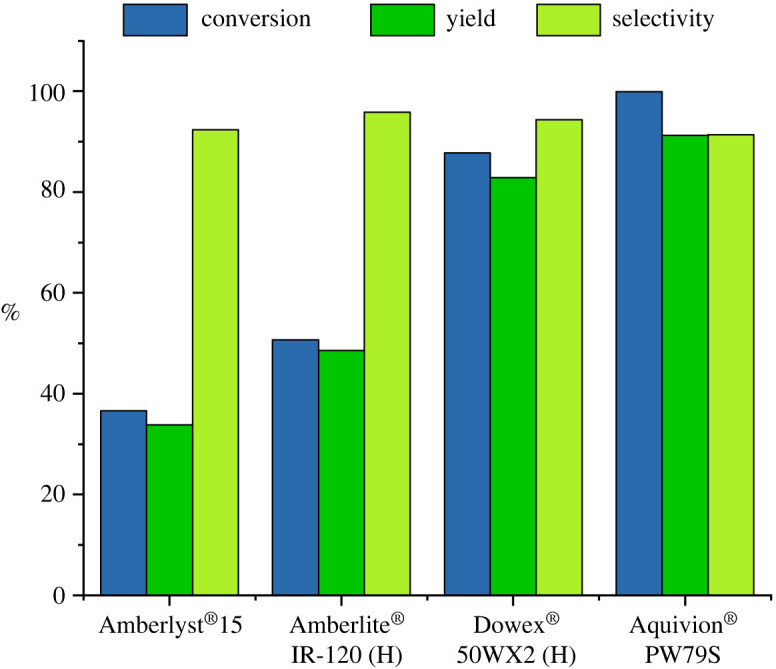
Comparison of conversion, yield and selectivity in the presence of $33\;\mathrm{meq}\;H^{+}$.

As a comparison, it is worth noting that using the same $H^{+}$ equivalent of the other investigated catalysts, we have obtained good selectivities but only moderate substrate conversions (table 1, entries 2, 5 and 9), as highlighted in the bar graph shown in figure 4. Effectively it is known that the main factor affecting the selectivity of this reaction is the $H^{+}$-catalysed epoxy ring opening side reaction. However, in the presence of ion exchange resins the bulky molecules of epoxidized triglycerides cannot enter the resin pores, even if the resins swell in an organic solvents [48]; thus only the active sites collocated in the external surface area, can catalyse the ring opening of epoxidized triglycerides, hence the selectivity is maintained. For this reason, it is mandatory to determine the lowest amount of catalyst necessary to accomplish the reaction, avoiding decrease in selectivity. Experiments with an incomplete conversion were selected to calculate the turnover frequency (TOF) of the different catalysts (table 1, entries 2, 5, 9 and 11). Overall, the result in table 1 and the bar graph in figure 4, show that Aquivion PW79S displays outstanding activity for the epoxidation of the safflower oil, leading to complete conversion into the desired product with the least amount of catalyst and above all the highest TOF values.

### 3.3. Reactivity of Aquivion and Dowex toward different oils and stability

With these results and to extend the scope of our work, we decided to investigate the catalytic activity of the most efficient catalysts Aquivion PW79S and Dowex® 50WX2 (H) toward the epoxidation of vegetable oils with a different composition of fatty acid chains including a WCO (table 2). Indeed, it is known that unsaturated fatty acid composition of vegetable oils affects the reactivity in the Prilezhaev epoxidation [49]. These experiments (table 2) showed the same reactivity for all the vegetable oils investigated, including the WCO, with the only exception of the hemp oil (table 2, entries 4 and 6) rich in tri-unsaturated alkyl chains. Despite what has been reported so far [49] for vegetable oils rich in tri-unsaturated fatty acids, hemp oil requires a double amount of both the catalysts to obtain quantitative conversion of the substrate (table 2, entries 4 and 6). *q* ^1^HNMR analysis also revealed that the double bond in the middle position is the least reactive one.

**Figure 5.**
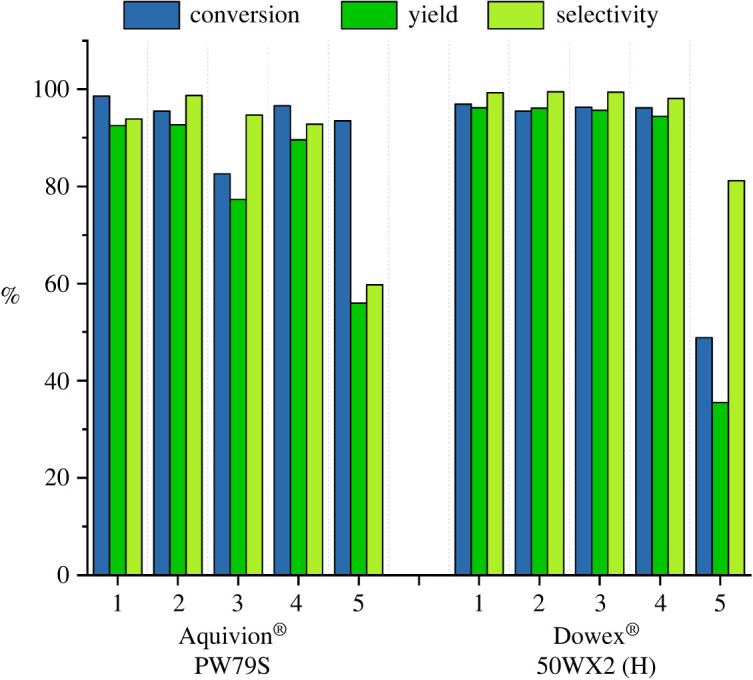
Conversion, yield and selectivity as a function of the number of reuses of catalysts (Aquivion and Dowex).

The stability of the more efficient catalysts Aquivion PW79S and Dowex® 50WX2 (H) was also evaluated by repeating five reaction cycles under optimized operational conditions. After each reaction, the resin was filtered, rinsed with ethyl acetate, dried under vacuum and employed again. These experiments revealed that either Aquivion PW79S and Dowex® 50WX2 (H) can be used four times without loss of their catalytic activity (figure 5).

## 4. Conclusion

The epoxidation of vegetable oils with different fatty acid composition was carried out with peracetic acid formed *in situ* from acetic acid and 30% hydrogen peroxide solution in the presence of AIER catalyst obtaining almost complete conversion and selectivity. Although the reactivity of the double bonds toward the epoxidation effectively depends upon the unsaturation degree, the proposed methodology allows the selective transformation of different vegetable oils into the corresponding epoxides with a high degree of purity. In this study, a commercial thermostable perfluorosulfonic acid (PFSA) resin, Aquivion PW79S was used for the first time to catalyse this reaction. Its catalytic performance, ease of recovery and deactivation rate were assessed with respect to the most used AIERs epoxidation catalysts. Probably its success can be ascribed to its unique combination of properties: amphiphilic superacid activity with the ability to stabilize Pickering-like emulsions, thermal and chemical stability, crystalline open structures, commercial availability with different pore sizes [29], which make it suitable in the selective epoxidation of vegetable oils. Finally, Aquivion PFSA, under our operational conditions, should not induce supplemental cost, as it is conveniently recovered by filtration and re-used without any treatment. Quantitative ^1^HNMR spectroscopy has been used, as robust methodology, to determine the chemical properties of triglycerides and epoxidates triglycerides as well as conversion, yield and selectivity in the epoxidation reactions.

## Data Availability

Data supporting this study have been uploaded as electronic supplementary material [50].
